# Application of omics technologies to identify reproductive phenotypes in the context of assisted reproductive technologies for cattle

**DOI:** 10.1590/1984-3143-AR2026-0084

**Published:** 2026-07-27

**Authors:** Rochelle Veldhuizen, Maria Belen Rabaglino

**Affiliations:** 1 Department of Population Health Sciences, Faculty of Veterinary Medicine, Utrecht University, Utrecht, Netherlands

**Keywords:** breeding, biomarkers, cattle production, reproductive potential

## Abstract

Fertility is a key determinant of reproductive efficiency, economic performance, and sustainability in cattle production systems. Traditional fertility phenotypes that are still widely used today, such as conception rate, calving interval, non-return rate, and semen quality parameters, primarily describe reproductive outcomes rather than predict fertility. Consequently, important molecular and physiological differences between animals with contrasting fertility may remain undetected. Omics technologies provide new opportunities to identify reproductive phenotypes that more accurately reflect the biological mechanisms underlying fertility in cattle. Genomics enables the identification of single nucleotide polymorphisms, quantitative trait loci, and haplotypes associated with variation in fertility. Transcriptomics has revealed gene expression patterns related to spermatogenesis, sperm function, uterine receptivity, and embryo-maternal communication. Proteomics has identified protein profiles associated with oocyte developmental competence, fertilization capacity, and embryonic development, while metabolomics reflects the biochemical state most closely linked to the expressed phenotype, identifying biomarkers associated with oxidative balance and energy, lipid, and protein metabolism. This manuscript reviews selected studies that have applied omics technologies to identify measurable reproductive phenotypes that better capture the biological basis of fertility in cattle within the context of assisted reproductive technologies. While genomics remains the most widely applied omics approach due to the stability and accessibility of DNA, downstream omics may provide a more accurate representation of the dynamic biological processes defining fertility. Future research should focus on translating these findings into practical assays for rapid and accessible biomarker measurement, supporting informed decision-making at the farm level.

## Introduction

Cattle are a core species in the global livestock industry and play an essential role in food security and economic development. Beef and dairy cattle contribute substantially to human nutrition by providing high-quality protein and dairy products to billions of people worldwide (Wang et al., 2025). In addition, cattle farming plays an important role in maintaining biodiversity, regulating soil processes, and supporting rural landscapes (Dumont et al., 2019). At the same time, the sector faces increasing social and regulatory pressure due to its significant contribution to greenhouse gas emissions, accounting for approximately 65% of total livestock-related emissions (Harindintwali et al., 2021). Notably, cattle production systems with lower productivity tend to exhibit higher emission intensities, primarily due to inefficiencies in feed conversion and herd performance. Therefore, strategies that increase productive output per animal represent one of the most effective approaches to reduce emission intensity (FAO, 2013). In this context, enhancing desirable genetic traits related to productivity and resilience, together with improving reproductive efficiency, have become central determinants for achieving sustainability in dairy and beef farm systems.

The development of assisted reproductive technologies (ART) in the 20th century enabled a deeper understanding of reproductive biology and facilitated the identification of key biological mechanisms underlying reproductive success or failure, making ART the most effective tools for improving genetics in cattle (Betteridge, 2003; Mapletoft and Hasler, 2005). The most widely used ART in cattle is artificial insemination (AI), which allows the dissemination of superior male genetics, as a single ejaculate can be divided into multiple doses and preserved for storage and global distribution. Another major technology is embryo transfer (ET), which enables the multiplication of genetic merit from both males and females. Embryos transferred into recipient or surrogate cows can be produced *in vivo*, within the female reproductive tract following Multiple Ovulation Embryo Transfer (MOET), or *in vitro*, under laboratory conditions through in vitro production (IVP) (Mapletoft and Hasler, 2005). While embryos derived after MOET were predominant when this technology expanded commercially in the 1990s, IVP embryos have surpassed them over the last decade, with more than 2 million IVP embryos transferred worldwide annually and current estimates indicating ~7-fold higher usage compared to in vivo embryos (Viana, 2025).

Because the primary objective of ART in cattle is genetic improvement rather than the treatment of infertility, animals included in these programs are expected to have sufficient baseline fertility to ensure optimal outcomes. Traditionally, fertility evaluation in cattle relies on phenotypic indicators reflecting observable reproductive performance. In bulls, fertility is typically assessed through breeding soundness examination, which includes parameters such as scrotal circumference, semen volume, sperm concentration, motility, and morphology. In cows, commonly used indicators include calving interval, non-return rate, days open, pregnancy rate, and conception rate. However, these conventional metrics do not directly capture the driving biological mechanisms of fertility, and important molecular differences between animals with variable fertility may remain undetected.

This limitation highlights the need for approaches that more accurately link reproductive phenotypes to their biological basis. A promising avenue lies in the application of omics technologies, which have become increasingly accessible in recent decades. Omics approaches enable comprehensive measurement of the molecular components defining a phenotype and can therefore be used to characterize the biological status of key tissues and cell types in both males and females.

The aim of this manuscript is to review studies that have applied omics technologies to help identify measurable phenotypes that better reflect the biological mechanisms underlying fertility in cattle, in the context of ART. Before delving into the omics technologies applied to males or females, a brief description of the main characteristics of each available omics approach will first be provided.

## Omics technologies

**Figure 1:**
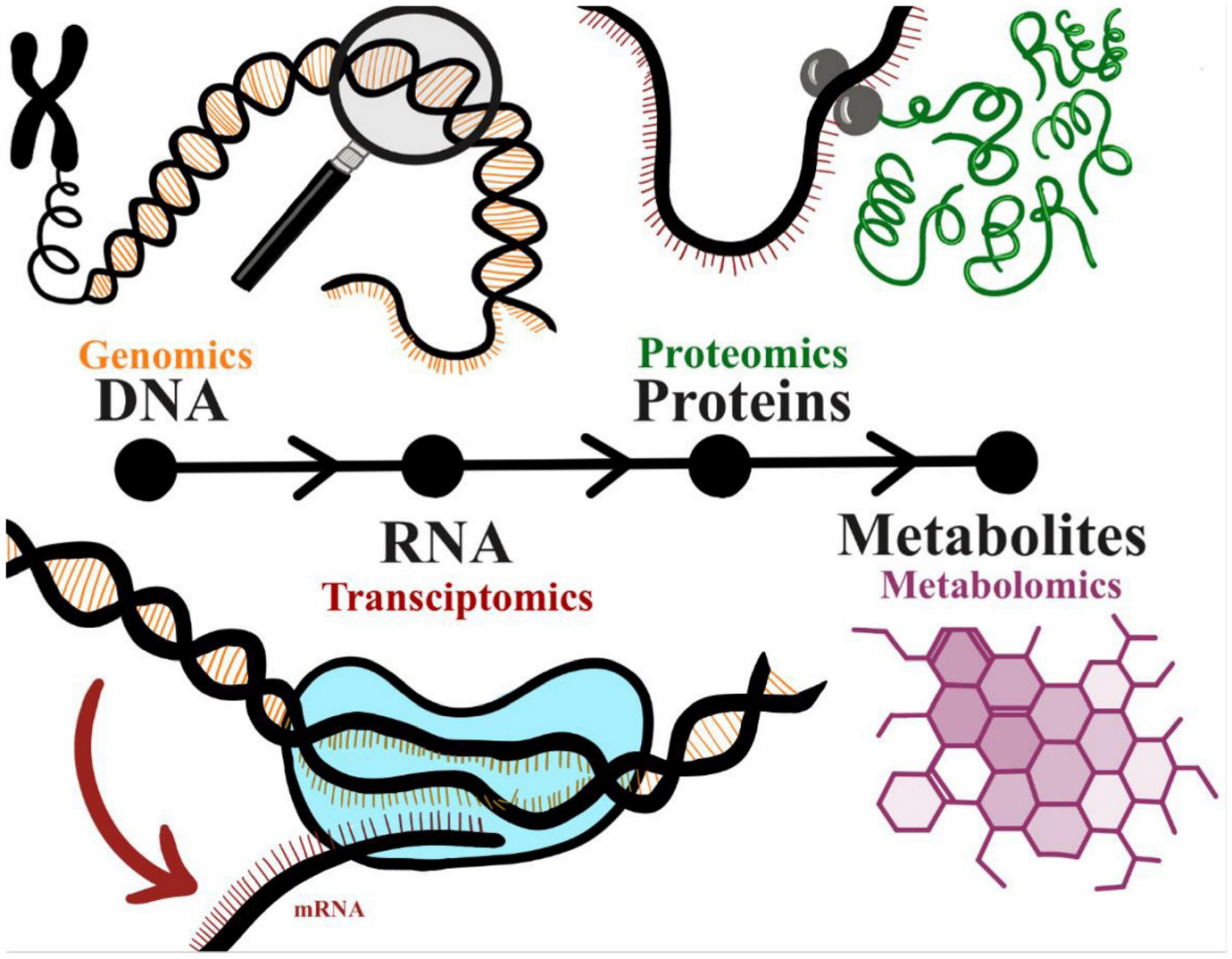
Representation of the omics technologies and the molecules they measure.

The use of high-throughput techniques has allowed not only the detection of qualitative differences but also the quantification of biomolecules that define a phenotype (Chakraborty et al., 2022; Hasin et al., 2017). Their improved sensitivity, specificity, and accuracy facilitate the rapid identification of biomarkers for biological characterization (Kim et al., 2017). The main omics approaches (Fig. 1) are discussed below.

### Genomics (“what can happen”)

The development of the polymerase chain reaction (PCR) in 1986 was a major milestone in molecular biology, enabling detailed investigation of DNA structure (Mullis and Faloona, 1987). Subsequently, advances in large-scale genome sequencing and assembly enabled the publication of the first bovine reference genome in 2009, which represented a significant advance in understanding ruminant biology and evolution (Bovine Genome Sequencing and Analysis Consortium, 2009). Later developments in next-generation and long-read sequencing technologies further improved genome mapping, variant discovery, and functional genomic analyses in cattle. Single nucleotide polymorphisms (SNPs) are variations in a single base pair within the genome, typically generating two alleles at a given locus, which may be associated with specific phenotypes. Genome-wide association studies (GWAS) are used to identify significant associations between molecular markers and phenotypic traits, often using commercial panels containing thousands of SNPs available for cattle. These approaches facilitate the identification of informative SNPs and improve the precision of genetic selection by linking molecular markers to complex traits, such as daughter pregnancy rate and cow/heifer conception rate (Berry et al., 2014).

### Transcriptomics (“what appears to be happening”)

The complete set of RNA transcripts, known as the transcriptome, can be characterized using transcriptomics. This high-throughput approach enables the analysis of gene expression dynamics and regulatory mechanisms involved in physiological processes and environmental adaptation (JiaYu et al., 2025). Early transcriptomic studies relied on microarray technologies, which are based on hybridisation, whereby synthetic single-stranded DNA or RNA probes bind to complementary sequences (Genomics Education Programme, 2021). However, microarray-based transcriptomic approaches are limited by their reliance on predefined probe sets designed from existing sequence knowledge, which limits their ability to detect new or unrepresented transcripts (Liu et al., 2007). RNA-sequencing (RNAseq) is currently the primary technology used in transcriptomics to map and quantify RNA molecules and is widely applied in basic research, clinical diagnostics, and drug development (Wang et al., 2009). RNAseq can detect gene polymorphisms and enable the direct measurement of isoforms or allele-specific expression, providing high-resolution transcriptome profiling. Due to its robustness and increasing accessibility, RNAseq has become the method of choice for global gene expression analysis (Corchete et al., 2020).

### Proteomics (“what makes it happen”)

Proteomics focuses on the study of protein composition, structure, function, and interactions within biological systems, providing insights into the functional machinery of the cell. However, proteomic analysis is inherently complex, as protein expression is highly dynamic and influenced by temporal and environmental factors. Over the past decade, proteomics has been broadly classified into two main areas: protein expression mapping and protein interaction mapping (Al-Amrani et al., 2021). Proteome analysis is typically performed using mass spectrometry, which identifies molecules by separating positively charged ions according to their mass-to-charge ratio. Protein identification is achieved through database search algorithms that match experimental mass spectra with reference protein or peptide sequences (Al-Amrani et al., 2021; Angel et al., 2012).

### Metabolomics (“what has happened”)

As the most downstream omics layer, metabolomics reflects the biochemical state most closely related to the expressed phenotype (Clish, 2015; Fiehn, 2002). It provides both quantitative and qualitative information on small-molecule metabolites and relies on high-throughput analytical techniques such as nuclear magnetic resonance, gas chromatography-mass spectrometry, and spectroscopy (Zhao et al., 2025b). These analyses reveal complex biological dynamics, enabling a better understanding of energy metabolism, redox balance, and amino acid turnover (Zhao et al., 2025a).

## Application of omics technologies to improve the outcomes of assisted reproductive technologies

Each of the omics technologies described above can be applied in assisted reproduction to identify the optimal molecular characteristics of key cells and tissues involved in reproduction. The application of omics enables the study of the main biological drivers of pregnancy establishment, facilitating the identification of molecular patterns associated with successful reproductive outcomes. Therefore, these approaches represent promising tools to improve the efficiency and effectiveness of ART (Egea et al., 2014). The main omics approaches used to characterize reproductive phenotypes in both bulls and cows are discussed below. An overview of each omics is summarized in Table 1.

**Table 1:** Overview of the main omics technologies and their applications to characterise fertility in (A) bulls and (B) cows.

**A) Bulls**				
*Omics*	*Sample measured*	*Phenotype (examples)*	*Functional impact*	*Application*
Genomics	DNA (blood, hair, semen)	SCR, semen quality, fertility-associated SNPs/QTL	Genetic variants affecting spermatogenesis, sperm function, fertilization	Genomic selection (GEBV), detection of lethal haplotypes, breeding decisions
Transcriptomics	Spermatozoa RNA (mRNA, miRNA)	Fertility index, ERCR, NRR	Regulation of spermatogenesis, sperm function, fertilization, early embryo development	Biomarker discovery for fertility prediction in AI programs
Proteomics	Seminal plasma, spermatozoa	Fertility index, fertilization outcomes	Proteins related to motility (axoneme), metabolism, capacitation, acrosome reaction, embryo development	Biomarker discovery for fertility prediction in AI programs, improvement of bull selection, optimization of IVP
Metabolomics	Seminal plasma, semen	Fertility index, sperm motility, oxidative status	Energy metabolism, lipid metabolism, redox balance, sperm function	Biomarker discovery, fertility prediction, semen quality assessment
**B) Cows**				
*Omics*	*Sample measured*	*Phenotype (examples)*	*Functional impact*	*Application*
Genomics	DNA (blood, hair)	Pregnancy rate, conception rate, embryonic loss	Polygenic control of fertility, genetic predisposition	Genomic selection, mate allocation, inbreeding management
Transcriptomics	Endometrium (cytobrush), granulosa cells, cumulus cells	Pregnancy outcome (AI/ET), endometrial receptivity, oocyte competence	Immune response, inflammation, embryo-maternal communication, oocyte development	Prediction of pregnancy, donor/recipient selection, IVP success
Proteomics	FF, UF, OF, EV, cervical mucus	Oocyte quality, embryo viability, fertility index	Follicular function, sperm-oocyte interaction, fertilization, embryo development, metabolic processes	Biomarker discovery, improvement of IVP systems, uterine environment assessment
Metabolomics	FF, UF, OF, serum	Oocyte yield, fertility index, receptivity	Energy metabolism, lipid metabolism, amino acid turnover, redox balance	Fertility prediction, oocyte competence assessment, optimization of ET

SNP, single nucleotide polymorphism; QTL, quantitative trait locus; GEBV, genomic estimated breeding value; mRNA, messenger RNA; miRNA, microRNA; SCR, sire conception rate; ERCR, estimated relative conception rate; NRR, non-return rate; AI, artificial insemination; ET, embryo transfer; IVP, in vitro production; FF, follicular fluid; UF, uterine fluid; OF, oviductal fluid; EV, extracellular vesicles.

## Male fertility: omics-defined reproductive phenotypes

### Genomics

GWAS in Holstein bulls have identified genomic regions associated with male fertility (Taylor et al., 2018). For instance, a GWAS study conducted in 1716 Irish Holstein bulls with different field fertility identified one SNP associated with pregnancy rate and 21 SNPs associated with semen quality (Abril-Parreño et al., 2023), while a GWAS in 1755 American Holstein bulls identified eight SNPs defining five quantitative trait loci that were related to sire conception rate (SCR) (Peñagaricano et al., 2012). Using a larger dataset encompassing the entire Holstein bull fertility evaluation in the United States by 2016 (44,449 SCR records), GWAS identified an additional six QTLs across multiple chromosomes, each explaining at least 0.5% of the additive genetic variance in SCR. Overall, SNPs were estimated to explain 32% of the phenotypic variance in SCR. Genes located within these regions were associated with sperm biology, including sperm development, fertilization, sperm motility, calcium channel regulation, and SNARE proteins (Han and Peñagaricano, 2016).

The combined effect of genome-wide SNP markers is captured as genomic estimated breeding value (GEBV), which is used in genomic selection. By incorporating thousands of DNA markers simultaneously, this approach aims to capture the majority of QTLs affecting a trait (Hayes et al., 2009). This allows early prediction of genetic merit in young animals without the need for progeny testing or extensive pedigree information (Habier et al., 2013; Hayes et al., 2009). Nevertheless, the prediction accuracy for SCR remains limited, which reflects the complex and polygenic nature of bull fertility (Abdollahi-Arpanahi et al., 2017).

Genomic technologies have also enabled the detection of recessive lethal haplotypes associated with embryo loss and reduced fertility (VanRaden et al., 2011; VanRaden and Miller, 2006). The combination of high-density SNP genotyping and homozygosity mapping has proven to be an efficient strategy for the rapid detection of novel genetic defects and the identification of underlying mutations, even when only a limited number of cases are available (Charlier et al., 2008; Lander and Botstein, 1987).

### Transcriptomics

Analysis of the sperm transcriptome, including both coding and non-coding RNA, provides insights into spermatogenesis and the role of sperm in fertilization and early embryo development. These molecular profiles can complement outcome-based indicators such as estimated relative conception rate and non-return rate (Indriastuti et al., 2022; Selvaraju et al., 2018). Several studies have identified differentially expressed mRNA transcripts and non-coding microRNA (miRNA) between spermatozoa from high-fertility (HF) and low-fertility (LF) bulls, associated with spermatogenic competence, sperm function, fertilization potential, and early embryonic development, supporting their potential use as biomarkers for bull fertility in AI programs (Capra et al., 2017; Donnellan et al., 2022; Indriastuti et al., 2022; Selvaraju et al., 2018; Turri et al., 2021).

### Proteomics

Proteomic profiling of seminal plasma and spermatozoa in bulls can help pinpoint molecular markers of sperm function, making it a valuable tool in andrological research (Wright et al., 2012). Differentially abundant proteins identified between HF and LF Holstein-Friesian bulls with divergent fertility in the field showed that proteins more abundant in HF bulls were mainly related to axoneme assembly and sperm motility, whereas those more abundant in LF bulls were related to metabolism, proteasome activity, and cell-cell recognition (Rabaglino et al., 2022). Furthermore, 34 biomarker proteins were selected using a machine learning approach, and their abundance was used to build a predictive model that classified HF and LF bulls from an external population with 94.4% accuracy.

Proteomics has also been applied to bull fertility in the context of in vitro embryo production. Bulls with similar sperm motility parameters but different fertilization outcomes exhibited significant proteomic differences in sperm and in the resulting blastocyst (Alomar et al., 2008; Ohgoda et al., 1988; Saeki et al., 1995). These differences were linked to key processes such as capacitation, the acrosome reaction, polyspermy, and the resulting blastocyst quality. This suggests that proteomics could improve selection of semen used for in vitro fertilization during IVP (Fernández-Montoro et al., 2025).

### Metabolomics

Metabolites present in seminal plasma or semen play essential roles in sperm function, including energy production, motility, protection, pH regulation, and control of metabolic activity (Bieniek et al., 2016). Because metabolites are end products of biochemical pathways and closely reflect phenotypic states, metabolomics may help identify biomarkers that more accurately capture the biological mechanisms defining bull fertility (Guijas et al., 2018; Kumar et al., 2015; Velho et al., 2018). Indeed, distinct metabolic patterns were found in the seminal plasma of Holstein bulls with varying *in vivo* fertility, indicating that seminal metabolites may serve as predictive biomarkers (Velho et al., 2018). Hoorn et al. (2026) identified differences in semen metabolism, particularly in lipid and energy pathways, associated with bull fertility. Moreover, a supervised machine learning approach designed to detect subtle multivariable differences revealed distinct metabolic signatures in sperm from HF and LF bulls, further supporting the potential of semen metabolomics for fertility prediction.

Metabolomics has also been applied to study oxidative stress, a key factor that can damage sperm cells and impair motility, membrane integrity, and fertilization capacity (Bollwein and Bittner, 2018). For instance, bulls with a higher total progressive motile sperm count had lower reactive oxygen species levels in their semen (Pang et al., 2025), and clear metabolomic differences and oxidative stress markers have been reported in high- and low-motility sperm samples (Blanco-Prieto et al., 2023; Giaretta et al., 2025; Vigolo et al., 2022).

## Female fertility: omics-defined reproductive phenotypes

### Genomics

Fertility in cows is a complex, multifactorial trait encompassing oestrous cyclicity, conception, embryo survival, maintenance of pregnancy, calving, and the resumption of cyclicity postpartum. These components typically exhibit low heritability and are strongly influenced by management practices and environmental conditions (Kadarmideen and Wegmann, 2003; VanRaden et al., 2004). Results from GWAS have generally been modest and difficult to replicate across populations, reflecting the highly polygenic architecture of fertility traits. While several genomic regions have been associated with fertility, loci on BTA18 are among the most consistently reported across studies (Brand et al., 2010; Dachs et al., 2023; Freebern et al., 2020). Overall, the small effect sizes and distributed genetic control limit the power of GWAS to identify robust, reproducible associations (Ma et al., 2019). Nevertheless, genomic selection has contributed to measurable improvements in dairy cattle fertility, demonstrating that genetic progress is achievable despite the biological and statistical complexity of these traits (VanRaden et al., 2004). For example, one study showed that using genomic rather than pedigree-based relationships improved mate selection by reducing expected inbreeding in the offspring while increasing expected progeny merit (Sun et al., 2013). Another study aimed to identify gene sets associated with the number of inseminations required for heifers to achieve pregnancy after AI (n = 2754) or ET (n = 1566). The authors identified 14 genes previously reported in fertility-related GWAS, in addition to 16 genes associated with embryonic loss (Kelson et al., 2025).

### Transcriptomics

Studies assessing the transcriptome in relation to female fertility must consider both the reproductive tissue and the specific stage of the cycle at which the molecular state is evaluated. Thus, the transcriptomic studies reviewed below are classified based on the cow's role within ART programs.

#### Cows on AI programs

During AI, sperm is deposited directly into the uterine body, bypassing the cervix. As a result, sperm first interacts with the endometrium rather than the vaginal epithelium, as occurs during natural mating. Therefore, the endometrial molecular profile plays a key role in sperm selection and capacitation (Holt and Fazeli, 2015). In a study involving 193 cows, endometrial samples collected via cytobrush immediately prior to AI revealed differences between cows that became pregnant and those that did not. A set of 57 biomarker genes, primarily involved in immune and inflammatory pathways, predicted pregnancy outcome with an average accuracy of 77% (Hoorn et al., 2024). In another study, endometrial samples collected 12 hours after AI with HF or LF bulls showed that the endometrium responded differently depending on the fertility status of the sire (Donnellan et al., 2023).

#### Cows on ET programs: donors

In donor cows, transcriptomics is primarily used to identify molecular markers associated with oocyte quality, fertility, and the efficiency of embryo production (Mazzoni et al., 2017; Uyar et al., 2013). Much of this research has focused on granulosa cells, which can be readily collected during oocyte aspiration and thus represent a practical, non-invasive source of biomarkers for IVP success (Mazzoni et al., 2017). Indeed, the transcriptome of cumulus cells has been shown to reflect oocyte developmental competence, as these cells maintain close paracrine communication with the oocyte (Macaulay et al., 2016, 2014; Zhang et al., 1995).

#### Cows on ET programs: recipients

Despite evidence supporting the benefits of uterine exposure to semen during natural mating or insemination (Chastant and Saint-Dizier, 2019), several studies indicate that the endometrium can prepare for embryo reception independently during each cycle. In virgin heifers, both the oviduct and the endometrium ipsilateral to the ovulatory ovary exhibit distinct transcriptomic profiles compared to the contralateral side as early as two days post-ovulation. Additionally, gene expression varies spatially along the reproductive tract, suggesting that intrinsic tract-specific mechanisms contribute to embryo survival (Hoorn et al., 2023). The endometrium is already responsive to embryonic signals by day 3 (Maillo et al., 2015), and the endometrial transcriptome at day 7, prior to embryo contact, differs between cows that subsequently establish pregnancy and those that do not (Mazzoni et al., 2020; Ponsuksili et al., 2012; Salilew-Wondim et al., 2010). Moreover, applying bioinformatics and machine learning to the integrated data from these studies has identified a set of 50 biomarker genes that predict endometrial receptivity with an accuracy of 96.1% (Rabaglino and Kadarmideen, 2020).

### Proteomics

The identification of proteins as potential fertility biomarkers has been achieved through proteomic analyses of follicular fluid (FF), uterine fluid (UF), and oviductal fluid (OF). In FF, eight proteins related to follicular function were found to be differentially abundant in samples from less fertile cows (repeat breeder cows) compared to controls (Zachut et al., 2016). Proteomic profiling of extracellular vesicles (EV) in OF, as well as in in vitro oviduct epithelial cell models, identified 186 differentially abundant EV proteins between *in vivo* and *in vitro* systems. Notably, several proteins associated with sperm-oocyte binding, fertilization, and embryo development were absent in the *in vitro* system, highlighting their potential relevance for improving IVP conditions (Almiñana et al., 2017). Regarding UF, uterine histotroph collected 7 days after AI was compared between cows that retained a viable embryo and those in which the embryo degenerated or arrested. One protein, PAFAH1B3, was detected exclusively in the histotroph of cows with viable embryos, suggesting its involvement in early embryonic development (Beltman et al., 2014). In another study, the UF proteome at day 19 of pregnancy was compared between Holstein heifers with a low or high fertility index, revealing that proteins more abundant in the LF group were associated with disrupted metabolic processes (Gegenfurtner et al., 2020). Although this study reflects consequences of fertility rather than predictive markers, proteins in cervical mucus collected prior to AI have been identified as potential biomarkers of fertility in Bali heifers (*Bos javanicus*) (Yusuf et al., 2026).

### Metabolomics

Similar to proteins, metabolites indicative of fertility traits have been measured in FF and UF. In a study conducted in *Bos indicus* cattle, several metabolic compounds in FF clearly discriminated donors classified according to oocyte yield: HF (>15 oocytes), medium fertility (5–15 oocytes), and LF (<5 oocytes). These metabolic markers achieved area under the curve (AUC) values ranging from 0.86 to 0.99 for distinguishing HF and LF donors (Guerreiro et al., 2018). Another study in Holstein heifers classified as Fert+ or Fert-, based on being in the top 20% or bottom 5% for calving interval genetic merit, identified differences in the abundance of several fatty acids in FF and serum, as well as amino acids in FF. These metabolites showed high predictive power for fertility genotype, as indicated by their AUC values (Moore et al., 2017). Metabolomic studies of UF have shown that its composition changes dynamically throughout the oestrous cycle, with metabolite concentrations peaking around days 5 to 7 post-ovulation (Tríbulo et al., 2019). In addition, the presence of an embryo alters the UF metabolomic profile (Sponchiado et al., 2019). These findings suggest that specific metabolites could serve as biomarkers of endometrial receptivity, particularly in the context of ET programs.

## Final considerations

Fertility in cattle is a complex, polygenic trait that has long been challenging to measure accurately. In bulls, fertility has been more extensively characterized due to the large volume of data generated through AI, which maximizes the use of each ejaculate. In contrast, data collection in cows remains slower and more limited, although the use of embryo technologies has enabled a greater quantification of reproductive outcomes. Nevertheless, there is still a need to better define the biological mechanisms driving fertility differences between individuals, in order to improve our understanding of reproductive success and potentially enable its optimization.

The development and increasing accessibility of omics technologies have opened new opportunities to address this challenge. As these approaches are more widely applied, they will enhance the identification of biologically meaningful reproductive phenotypes and improve our understanding of fertility at the molecular level. It should be noted that an important omics layer not discussed in this review is epigenomics, which refers to heritable changes in gene activity or function without altering the DNA sequence. One of the best-characterized epigenetic mechanisms is DNA methylation. Previous studies have shown that the sperm DNA methylome is associated with bull fertility (Kropp et al., 2017; Zhang and Sirard, 2025), and that DNA methylation patterns in immune cells are associated with fertility in postpartum cows (Bouzeraa et al., 2025), suggesting that epigenomics may be an emerging valuable tool for improving genetic traits in cattle breeding (Wang et al., 2023).

Among the omics approaches discussed, genomics remains the most widely applied. Its development has been considered a major breakthrough in cattle breeding, and ongoing advances continue to establish genomics as a practical and widely accessible tool for farmers (Ghavi Hossein-Zadeh, 2024). This widespread adoption is largely due to the properties of DNA, which is stable and present in all tissues, allowing for easy sampling from sources such as blood or hair. As highlighted in this review, selected studies have demonstrated the successful use of genomics to identify markers associated with fertility traits in both bulls and cows. However, genomics also has important limitations. As the first layer in the molecular cascade, it reflects “what could happen” rather than “what is happening.” Although genetic variants are heritable, the polygenic nature of fertility traits and their strong environmental modulation limit the predictive accuracy of genomics alone. Consequently, genomic studies typically require large populations to detect statistically significant associations. In addition, SNP-based analyses rely on discrete, static variation, focusing on the presence or absence of genetic markers, whereas other omics approaches, such as transcriptomics, proteomics, and metabolomics, capture continuous and dynamic biological responses. These downstream omics layers may therefore provide a more accurate representation of the biological processes contributing to fertility. However, their dynamic nature also represents a limitation, as biomarker measurements may need to be repeated over time rather than assessed once in an animal’s lifetime. Future developments in these fields should aim to translate molecular discoveries into practical, user-friendly tools. For example, rapid assays based on the expression of key biomarker genes from minimally invasive samples, such as cervical swabs, could help identify cows with a higher probability of pregnancy in a given cycle following AI or ET. Finally, future research should consider integrating information from both sides of the reproductive interaction. Evaluating molecular markers in semen and the peri-ovulatory endometrium in AI, or in the embryo and the day 7 endometrium in ET, may enable the identification of optimal biological matches, thereby maximizing the likelihood of successful pregnancy establishment.

## Data Availability

No research data was used.
